# Advances in the genetics of stroke risk and recovery

**DOI:** 10.1007/s00415-022-11525-w

**Published:** 2022-12-22

**Authors:** Sabbiha Nadia Majumder, James Hrastelj, Neil P. Robertson

**Affiliations:** 1 Department of Neurology, Morriston Hospital, Heol Maes Eglwys, Swansea, SA6 6NL UK; 2 Department of Neurology, Division of Psychological Medicine and Clinical Neuroscience, Cardiff University, University Hospital of Wales, Heath Park, Cardiff, CF14 4XN UK

Cerebrovascular disease (CVD) is widely considered a heterogeneous clinical syndrome rather than a single disorder. A large number of modifiable and non-modifiable risk factors have been associated with CVD, but even after accounting for these, there remains a large proportion of unexplained individual risk. Although genetic risk has commonly been considered non-modifiable, it is now better understood how environmental influences can modify gene expression as a result of epigenetic phenomena. Genome-Wide Association Studies (GWAS) have identified some genetic risk loci for stroke, many of which are common to CVD in general. It is hoped that a more comprehensive understanding of genetic risk in differing populations will have the potential to improve our understanding of disease pathogenesis and to identify novel therapeutic mechanisms. This in turn could be used to quantify risk of stroke at a young age, integrate with advice on lifestyle choices, and develop opportunities to target preventive treatments.

Two of this month’s papers focus on the genetic aetiology of stroke and stroke subtypes in different populations. The final paper examines the genetic basis of early neurological deterioration following ischemic stroke.

## Genome-wide association study meta-analysis of stroke in 22,000 individuals of African descent identifies novel associations with stroke

This genome-wide association meta-analysis focused on African ancestry populations with high stroke burden and identified novel genetic association for ischemic stroke. This was part of the consortium of minority population genome-wide association studies of stroke. It pooled data from 13 studies to compare the genomes of more than 22,000 black people (3734 cases, 18,317 controls). Keene et al. identified a variant located in close vicinity to a gene called *HNF1A* on chromosome 12 with genome-wide significance. *HNF1A* has been previously implicated in lipid metabolism and production of C-reactive peptide, and linked to the risk of developing diabetes, coronary artery disease, and stroke. A further 29 variants demonstrated association and fell within 24 distinct loci within the genome. 16 out of 24 unique loci were associated with stroke risk in individuals of European or Hispanic descent using stringent Bonferroni correction. In addition, variants with suggestive significance were identified in the *SFXN4* and *TMEM108* genes and may be novel ischemic stroke loci.

*Comment*: This study examined genetic stroke risk of populations who suffer stroke three times more frequently than other populations. Although the sample size is relatively small, it identified a genome-wide significant variant and demonstrates the importance of genetic research in marginalised populations.

Keene et al. (2020) Stroke 51:2454–2463.

## Genetic basis of lacunar stroke: a pooled analysis of individual patient data and genome-wide association studies

Recent GWAS have clearly demonstrated the genetic predisposition to specific subtypes of ischemic stroke. Large artery atherosclerotic stroke and cardio-embolic stroke share similar genetic risk factors to atherosclerosis and atrial fibrillation, respectively. However, the genetic basis of lacunar infarcts is less well understood despite accounting for about 20% of all ischemic strokes and being associated with long-term disability and dementia. Previous GWAS have only identified one genetic locus (16q24), in contrast with the 35 identified for large artery stroke and its major subtypes. Traylor et al. report a multi-trait meta-analysis GWAS of 2987 new MRI-confirmed cases of lacunar stroke plus 7338 from existing GWAS, compared with 254,798 controls. Meta-analysis was performed after dividing the study population into European ancestry and other ancestry. Five loci were found to be associated with lacunar infarct and a further seven were found to be associated with MRI features of lacunar infarct, such as cerebral white matter hyperintensities. Two of the novel loci contained genes that cause monogenic lacunar stroke. Expression levels of six genes were found to be associated with lacunar stroke by transcriptome-wide association study. Cardiovascular risk factors, including hypertension, smoking, and diabetes, were found to be associated with lacunar infarct by Mendelian randomisation.

*Comment*: The study included the largest number of cases of lacunar infarct with MRI confirmation to date. Overall, the results implicate disruption of the extracellular matrix, pericyte differentiation, TGFβ signalling, and myelination in lacunar stroke. Further investigation is warranted to elucidate how these processes contribute to lacunar stroke.

Traylor et al. (2021) Lancet Neurol 20: 351–61.

## Multi-ancestry GWAS reveals excitotoxicity associated with outcome after ischemic stroke

Whilst there are now more than 30 genetic loci associated with stroke risk, there has been relatively little progress in identifying genetic modifiers of stroke severity and recovery. Early deterioration in neurological deficit has been shown to affect long-term motor recovery following ischemic stroke. Ibanez et al. performed a multi-ancestry meta-analysis in 5876 acute ischemic stroke patients from seven countries to identify genetic variants associated with degree of early neurological disability. The difference in National Institute of Health Stroke Scale score within 6 h of symptom onset and at 24 h was used as a quantitative phenotype. First, a GWAS was performed in each country separately, except USA where the population was divided into European and African cohorts. In the second stage, a fixed-effect meta-analysis was performed among the same groups. Third, multi-ancestry Bayesian meta-analyses were performed. The analyses identified eight loci associated with early neurological deterioration with genome-wide significance. The loci explained 1.8% of the total variance. Summary data-based Mendelian randomisation and expression quantitative trait locus mapping indicated that *ADAM23* was driving association at one of the identified loci. Gene-based analysis implicated *GRIA1* as the most relevant gene at another associated locus. *ADAM23* is a presynaptic protein and *GRIA1* is a protein subunit of the AMPA receptor. Both are enriched in neurones and are responsible for modulation of neuronal excitability.

*Comment*: This study demonstrated a novel association between excitotoxicity and early neurological deterioration following ischemic stroke among multiple ethnic populations. The mechanisms by which excitotoxicity contributes to cell death may provide targets for neuroprotective therapies in early stroke.

Ibanez et al. (2022) Brain 145; 2394–2406.

